# Quality Assessment and Application Scenario Analysis of AGRI Land Aerosol Product from the Geostationary Satellite Fengyun-4B in China

**DOI:** 10.3390/s24165309

**Published:** 2024-08-16

**Authors:** Nan Wang, Bingqian Li, Zhili Jin, Wei Wang

**Affiliations:** School of Geosciences and Info-Physics, Central South University, Changsha 410083, China; wangnan2021@csu.edu.cn (N.W.); sharonli@csu.edu.cn (B.L.); jinzhili@csu.edu.cn (Z.J.)

**Keywords:** AOD, FY-4B, evaluation of AOD retrieval results, application prospects

## Abstract

The Advanced Geostationary Radiation Imager (AGRI) sensor on board the geostationary satellite Fengyun-4B (FY-4B) is capable of capturing particles in different phases in the atmospheric environment and acquiring aerosol observation data with high spatial and temporal resolution. To understand the quality of the Land Aerosol (LDA) product of AGRI and its application prospects, we conducted a comprehensive evaluation of the AGRI LDA AOD. Using the 550 nm AGRI LDA AOD (550 nm) of nearly 1 year (1 October 2022 to 30 September 2023) to compare with the Aerosol Robotic Network (AERONET), MODIS MAIAC, and Himawari-9/AHI AODs. Results show the erratic algorithmic performance of AGRI LDA AOD, the correlation coefficient (R), mean error (Bias), root mean square error (RMSE), and the percentage of data with errors falling within the expected error envelope of $\pm (0.05+0.15\times {\mathrm{AOD}}_{AERONET})$ (within EE15) of the LDA AOD dataset are 0.55, 0.328, 0.533, and 34%, respectively. The LDA AOD appears to be overestimated easily in the southern and western regions of China and performs poorly in the offshore areas, with an R of 0.43, a Bias of 0.334, a larger RMSE of 0.597, and a global climate observing system fraction (GCOSF) percentage of 15% compared to the inland areas (R = 0.60, Bias = 0.163, RMSE = 0.509, GCOSF = 17%). Future improvements should focus on surface reflectance calculation, water vapor attenuation, and more suitable aerosol model selection to improve the algorithm’s accuracy.

## 1. Introduction

Atmospheric aerosol refers to a general term for fine solid particles and liquid particles in the atmosphere, which are mainly derived from natural aerosols and bioaerosols such as fog, mist, and soot produced by nature, as well as anthropogenic aerosols caused by human activities like combustion, industrial emissions, and other sources [1,2]. Aerosols are directly or indirectly involved in the earth’s gas cycle process, act on the scattering and absorption of solar radiation, pose threats to human health, and significantly impact air pollution as well as the earth’s radiation balance [3,4,5,6]. Aerosol Optical Depth (AOD), as a vital aerosol optical properties parameter, indicates the vertical integration of the extinction coefficient of aerosols over the entire layer [7].

The Aerosol Robotic Network (AERONET), which relies on solar photometer measurements, is a crucial ground-based aerosol monitoring network that provides global, long-term, reliable observations of the optical properties of multispectral aerosols, including AOD [8,9]. However, the limited number and distribution of ground-based stations cannot meet the demand for monitoring aerosol temporal and spatial changes on large spatial scales. Satellite remote sensing technology, with its wide spatial and temporal coverage and various resolutions, effectively addresses this deficiency and has become an essential tool for monitoring the dynamics of aerosol optical thickness [10]. Examples include the GF-1 [11], FY-3 [12], HJ-1 [13], Landsat [14], VIIRS [15] satellites, and the Terra and Aqua satellites equipped with a Moderate Resolution Imaging Spectroradiometer (MODIS) sensor [16,17].

However, polar-orbiting satellites are limited by the revisit period, resulting in low temporal resolution, especially in the middle or lower latitude areas [18,19]. This limitation makes it difficult to meet the needs of emergency relief and disaster response, as these satellites cannot provide timely local observation data during sudden atmospheric pollution events. In contrast, geostationary orbit satellites have the advantage of high temporal resolution and can continuously observe the study area with real-time data updates. This capability effectively compensates for the low timeliness of polar orbit satellites.

China’s rapid economic development in recent years has been accompanied by the threat of air pollution, making the long-term and real-time monitoring of atmospheric pollution a critical goal [20]. Fengyun-4B (FY-4B) is China’s second-generation geostationary meteorological satellite, which carries the Advanced Geostationary Radiation Imager (AGRI), which is capable of capturing particles in different phases in the atmospheric environment and acquiring aerosol observation data with high spatial and temporal resolution. The AGRI has been applied to the study of aerosol retrieval algorithms [18,21,22]. The multichannel (MC) algorithm applied in the South Asia region has high accuracy. The accuracy validation assessment using 11 AERONET sites shows the statistical parameters of the MC AOD are better than those of the MODIS-combined DT and DB AOD datasets (RMSE = 0.16 of the MC AOD versus RMSE = 0.18 of MODIS) [18]. The results obtained by Jiang et al. [21] using the band ratio library (BRL) algorithm demonstrate the capability to represent the spatiotemporal distribution of AOD. This dataset is validated against AERONET data from June 2020, showing a good agreement with R is 0.84 and RMSE is 0.16. The AOD datasets retrieved by the land general aerosol (LaGA) algorithm show high consistency with the AERONET measurements (R > 0.83) [22].

In addition, the National Satellite Meteorological Center (NSMC) has announced the official Land Aerosol (LDA) product of AGRI; however, there is a lack of accuracy in the validation of this product. Therefore, this study uses AERONET ground-based monitoring data to validate the accuracy of the AGRI LDA product. AERONET measurements are highly accurate [8,9], making them a reliable ground truth for evaluating remote sensing products. This study assesses the performance of the AGRI LDA product by evaluating its algorithm performance across different spatial and temporal scales, as well as its dependence on angles, aerosol types, and surface types. Additionally, the reliability of AGRI LDA AOD results is compared with aerosol products derived from the MODIS Multi-Angle Implementation of Atmospheric Correction (MAIAC) AOD and Himawari-9/AHI L2 AOD.

## 2. Data and Methods

### 2.1. FY-4B LDA

Fengyun-4B (FY-4B) was launched on 3 June 2021, with a spatial resolution of 0.5–4 km. The satellite is equipped with the Advanced Geostationary Radiation Imager (AGRI) sensor, which contains 15 spectral channels ranging from 0.45 to 13.6 μm and is capable of conducting comprehensive disk observations every 15 min [18]. Additionally, it performs 165 regional observations across China on a daily basis, facilitating the rapid acquisition of aerosol observations.

FY-4B/AGRI provides Level 2 land aerosol products based on the dark target method, offering aerosol information for the 0.47, 0.55, and 0.65 µm channels, including AOD and Ångström Exponent (AE). These products cover the period from 20 August 2022, to 31 January 2024. This study evaluates the reliability of the LDA AOD at the 0.55 µm channel in China from October 2022 to September 2023 using ground-based AERONET aerosol measurements. The LDA data are available for free on the National Satellite Meteorological Center website (http://satellite.nsmc.org.cn/, accessed on 5 March 2024).

### 2.2. Evaluation Datasets

#### 2.2.1. Himawari-9/AHI

The Advanced Himawari Imager (AHI) sensor on the Himawari-8/9 geostationary satellites can perform full-disk observations every 10 min and regional observations every 2.5 min, with very high radiometric, spectral, and spatial resolution [23,24,25]. The AHI sensor has 16 spectral channels ranging from 0.43 µm to 13.4 µm, with a spatial resolution of 0.5 to 2 km. The Himawari-9/AHI official aerosol products were released since 7 July 2015 by the Japan Aerospace Exploration Agency (JAXA), providing information about AOD, optical thickness ratio, AE, and Quality Assessment (QA) flags. The products offer five channels (0.47, 0.51, 0.64, 0.86, and 1.61 µm) of land aerosol optical properties based on the retrieval algorithm designed by Fukuda et al. [26] and Higurashi and Nakajima [27]. A detailed description of the inversion algorithm can be found in Yoshida et al. [28]. In this study, the Level 2 aerosol products with a 10-min temporal resolution and a 5-km spatial resolution for the China region spanning the period from 1 October 2022, to 30 September 2023, were selected and downloaded from the P-Tree system website of the JAXA (https://www.eorc.jaxa.jp/ptree/, accessed on 29 March 2024).

#### 2.2.2. MODIS

**Figure 1:**
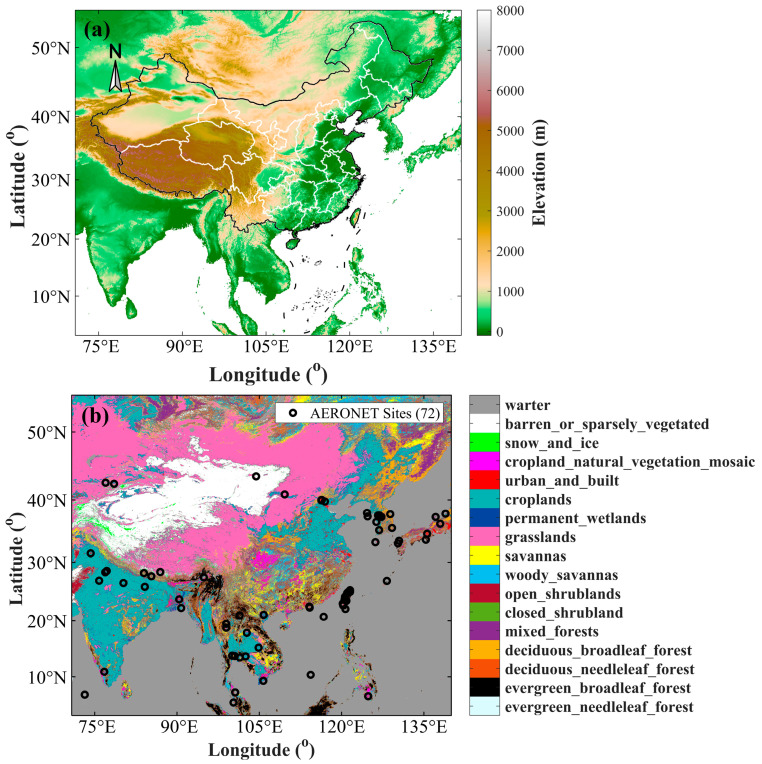
The terrain, land type, and distribution of AERONET sites in the study area: (**a**) topographic maps; (**b**) land type; and AERONET site distribution.

The MODIS Land Cover Type product (MCD12Q1) provides five global land cover classification schemes annually at 500 m SIN grid resolution for the years 2001–2022. This study adopts the International Geosphere-Biosphere Programme (IGBP) [29] global vegetation classification scheme, which identifies 17 land cover types worldwide [30,31]. The MCD12Q1 product is used to evaluate the accuracy of AGRI LDA AOD across different land use types. The terrain, land type, and distribution of AERONET sites in the study area are shown in Figure 1.

The MOD13Q1/MYD13Q1 dataset, a Level 3 product from MODIS Terra/Aqua, offers a spatial resolution of 250 m and includes two primary vegetation indices: NDVI and EVI [32,33]. In this study, MODIS NDVI data are utilized to conduct bias analysis in LDA AOD, specifically examining how surface cover influences LDA AOD bias.

MCD19A2 is a secondary daily AOD product provided by MODIS. The MCD19A2 product has a spatial resolution of 1 km and uses the MAIAC algorithm [34,35] for atmospheric corrections and aerosol retrievals, and its reliability in the Chinese region has been confirmed [36]. Since MCD19A2 MAIAC AOD is derived from Terra and Aqua satellites, the observation time of MAIAC AOD is set at 12:00 AM, and the MAIAC AOD has been involved in the accuracy analysis study of LDA AOD in this paper.

#### 2.2.3. AERONET

The AERONET site data is the aerosol observation network jointly organized by NASA and PHOTONS, and the ground-based sites of AERONET are distributed all over the world, which can realize global aerosol monitoring with high accuracy. In this paper, the AERONET level 1.5 AOD from 72 stations located in China and neighboring regions is used as the accuracy validation of the LDA AOD. Furthermore, considering that AERONET does not provide AOD at 0.55 µm, for consistency in comparison, this study calculates the AOD at 0.55 µm using AOD values from adjacent channels at 0.44 µm and 0.675 µm, following the conversion method described in Equation (1) [18,37]. In addition, the AERONET AE and SSA products were used to divide aerosol types into six types [38,39]. Simultaneously, the solar zenith angle, water vapor content, ozone content, nitrogen dioxide (NO_2_) content, SSA, and AE measured by AERONET are used for error and dependence analysis of LDA AOD.

(1) $$\tau_{0.55}=\tau_{0.44}\times {\left(0.55/0.44\right)}^{\frac{\ln\left(\tau_{0.44}/\tau_{0.55}\right)}{\ln\left(0.44/0.675\right)}}$$

where, $\tau_{0.44}$, $\tau_{0.55}$ and $\tau_{0.675}$ stand for the AOD of 0.44 µm, 0.55 µm, and 0.675 µm.

The LDA AOD values are averaged within a 30-km radius of each AERONET station to match the AERONET AOD. AERONET observations within a 1-h window centered on the LDA AOD measurement time are also averaged to match the LDA AOD. Averaging within a 30 km radius can mitigate the impact of chance errors on comparison results and reduce errors of spatial heterogeneity, thereby enhancing the spatial representativeness and statistical stability of the data.

### 2.3. Methods

In this study, AERONET measurements were employed to validate LDA AOD. To match the spatial resolution of LDA AOD, both MAIAC AOD and AHI AOD data were resampled to a spatial resolution of 4 km, with the temporal matching scale set to 1 h. Additionally, the matching temporal and spatial scales for the LDA AOD, MAIAC AOD, and AHI AOD datasets with AERONET measurements were standardized to 1 h and 30 km, respectively. The LDA AOD products are evaluated using the following statistical parameters:

(1) N: The number of samples, i.e., the number of point pairs matched between the FY-4B LDA AOD and the AERONET ground-based data;
(2) Correlation coefficient (R): The level of data correlation between the results to be validated and the ground-based data;
  (2) $$R=\frac{\sum_{i=1}^{N}\left(V_{i}-\bar{V_{i}})(V_{i}^{\prime }-\bar{V_{i}^{\prime }}\right)}{\sqrt{\sum_{i=1}^{N}{\left(V_{i}-\bar{V_{i}}\right)}^{2}{\left(V_{i}^{\prime }-\bar{V_{i}^{\prime }}\right)}^{2}}}$$
  where, N is the total number of matchups, $V_{i}$ denotes the AOD value for the ith sample of the AGRI, $V_{i}^{\prime }$ denotes the true value of the foundation measurement corresponding to $V_{i}$.
(3) Mean Error (ME, Bias): The average value of the difference between the data to be validated and the ground-based measurements. The mean error reflects the volatility of the data. A smaller ME indicates that the error is more evenly distributed across the entire dataset, indicating more reliable results;
  (3) $$ME(bias)=\frac{1}{N}\sum_{i=1}^{N}\left(V_{i}-V_{i}^{\prime }\right)$$
(4) Root Mean Square Error (RMSE): The degree of difference between the data to be validated and the measurements; the smaller the value of RMSE, the smaller the difference between the values to be validated and the measured values of the site;
  (4) $$RMSE=\sqrt{\frac{1}{N}\sum_{i=1}^{N}{\left(V_{i}-V_{i}^{\prime }\right)}^{2}}$$
(5) Expected Error (EE15): EE15 is a widely used evaluation parameter to describe the overall performance of satellite aerosol versus ground-based measurements and stands for the degree of data correlation between the satellite datasets and the measurements [40]. Generally, the satellite retrieval results to be validated are considered to have a good match with the AERONET measurements when at least 67% of the satellite results fall within the range of $\pm (0.05+0.15\times {\mathrm{AOD}}_{AERONET})$ (EE15) [41];
(6) EA ± EP(EE): Expected Accuracy (EA) is the best linear fit of the ground-based measurements to the mean deviation, indicating the degree of fluctuation of the mean deviation of the values to be validated from the true values. Expected Precision (EP) indicates the best linear fit to the one-fold standard deviation of the deviation, indicating the degree of fluctuation of the deviation between the value to be validated and the true value. The Expected Error (EE), defined as EA ± EP, is a function of the AERONET measurements, expressed in the notation of Huang et al. [42]. It is considered to meet the accuracy requirement if at least 68% of the data to be validated falls within the EE envelope when the satellite retrieval results are validated against AERONET;
(7) Global Climate Observing System Fraction (GCOSF): The Global Climate Observing System (GCOS) stipulates that the accuracy of satellite-retrieved AOD values should be better than 0.03 or 10% when compared to true values [43]. The GCOSF represents the overall percentage of AOD products that meet the requirements of GCOS [44]. The specific calculation principles are shown in the following formula.
  (5) $$\mathrm{GCOSF}=Max\left(0.03,0.1*AOD_{AEROENT}\right)$$

## 3. Results and Analysis

### 3.1. Validation by AERONET

#### 3.1.1. Overall Validation

**Figure 2:**
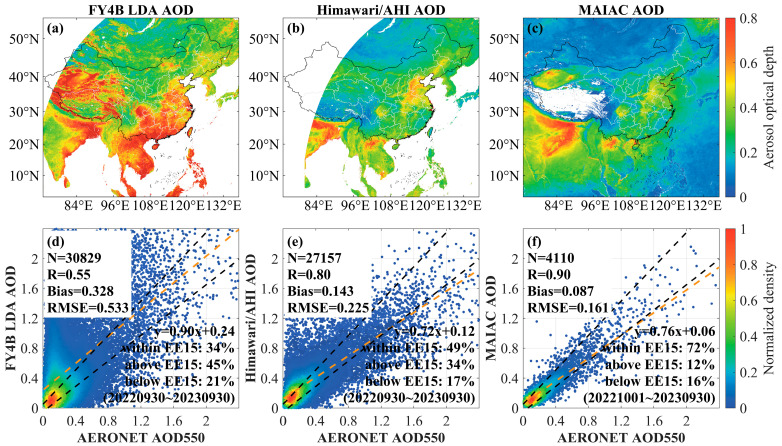
Overall distribution and validation scatter plots: (**a**) LDA AOD, (**b**) AHI AOD, (**c**) MAIAC AOD, and (**d**–**f**) Scatter plots of AGRI LDA AOD, AHI AOD, and MAIAC AOD compared with AERONET measurement data, respectively. The orange dashed line represents the linear fit between the ground-based measurements and the AOD dataset need be validated. The black dashed line indicates the envelope of the expected error (EE15).

To assess the reliability of the official AGRI LDA products, the 550 nm LDA AOD from 30 September 2022, to 30 September 2023, was selected and validated against the Himawari-9/AHI official AOD products and the MODIS MAIAC AOD products in this work. The total validation results for around one year between three AOD datasets and AERONET measurements are shown in Figure 2.

Figure 2a–c shows the spatial distribution of the annual mean values of the AGRI LDA, AHI AOD, and MODIS MAIAC AOD products in China. The results indicate that the LDA AOD is significantly overestimated in the southern coastal area and the western plateau area. Based on numerous experimental results, it is hypothesized that this overestimation phenomenon is related to the incomplete removal of water vapor during the retrieval process.

Figure 2d–f presents the scatter plots of three AOD datasets matched with ground-based sites, respectively. The matched samples with AERONET AOD are 30,829, 27,157, and 4110 pairs, with correlation coefficients of 0.55, 0.80, and 0.90, respectively. The AGRI LDA AOD shows a poor correlation with the AERONET measurements (R = 0.55), and there is a bigger bias in the study area (Bias = 0.328, RMSE = 0.533). The orange dashed line represents a linearly fitted straight line between the ground-based site data and the mean bias in the AOD dataset being validated. The black dashed line indicates the envelope of the expected error (EE15). The results show that 49% of the AHI AOD, 72% of the MODIS MAIAC AOD, and 34% of the AGRI LDA AOD matching points fall within the expected error envelope (EE15).

#### 3.1.2. Regional Validation

In this study, AOD datasets from 72 AERONET stations in China and nearby regions were selected to validate AGRI LDA AOD. Since the technical documentation of LDA AOD has not been publicly released and the land surface near the coastline is more susceptible to the influence of marine aerosols, this study classifies AERONET sites and land areas based on the minimum latitude and longitude distance (3 km) from the coastline. This classification aims to discuss and assess potential factors affecting algorithm accuracy.

Regions and sites are classified based on the smallest latitude and longitude distance (3 km) from the coastline. Land within 3 km of the coastline is categorized as an inland area, while land beyond 3 km is classified as a coastal area. Similarly, stations located within 3 km of the coastline are classified as inland stations, while those beyond 3 km are classified as coastal stations. The 72 stations were divided into 50 inland and 22 coastal stations, and the accuracy of the AOD datasets was validated and analyzed separately for the different regions.

**Figure 3:**
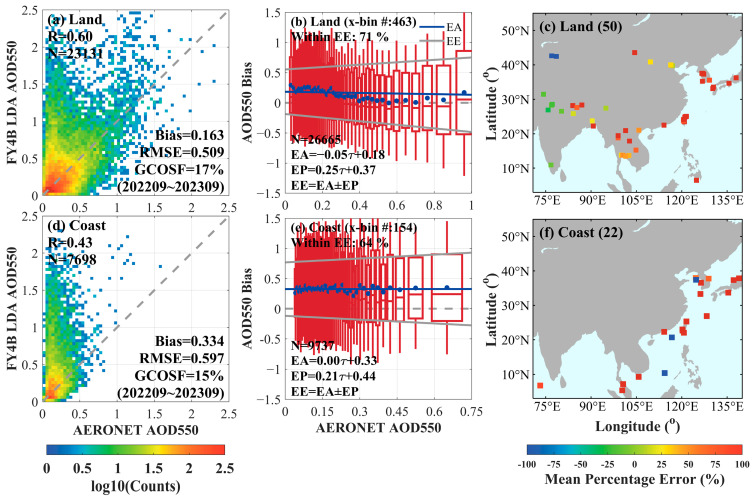
Validation results of LDA AOD and AERONET observations in inland and offshore areas. (**a**) Scatter plot for inland region validation; (**b**) Box plot for inland region error distribution; (**c**) Distribution of average percentage error for inland region; (**d**) Scatter plot for offshore region validation; (**e**) Box plot for offshore region error distribution; and (**f**) Distribution of average percentage error for offshore region.

The results of the validation of the LDA AOD with 50 inland sites and 22 offshore sites are presented in Figure 3. Figure 3a–c represents the validation scatter plots, box plots of the distribution of the AOD bias, and the mean percent error for the inland region for the LDA AOD and AERONET site data, respectively. Figure 3d–f shows the validation scatter plot, box plots of AOD deviation distribution varies with the AERONET measurements, and mean percentage error for LDA AOD versus AERONET data in the offshore region, respectively. As seen from the plots, overestimation of LDA AOD occurs in both inland and offshore regions. LDA AOD shows overestimation across all aerosol concentrations, especially in low-concentration and high-concentration aerosol regions. The scatter plots in Figure 3a,d indicate that the accuracy of LDA AOD is slightly worse in the offshore region compared to the inland region (R = 0.43, Bias = 0.334, RMSE = 0.597, GCOSF = 15%).

In Figure 3b, the LDA AOD bias was divided into 50 AERONET AOD intervals, each containing an equal number of samples. Approximately 463 sample pairs per interval were present in the inland region, while about 154 pairs were in the offshore region. The EA (blue solid line) in the figure represents the equation of the linear fit line (blue solid circle) to the mean deviation of the LDA AOD from the AERONET inland site data: −0.05 × τa + 0.18. The EP represented the fit to the linear regression of the one-fold standard deviation: 0.25 × τa + 0.37, which is shown in the red box. The uncertainty-range error envelope (EE) for the LDA AOD, represented by the EA ± EP (two gray lines), was obtained as [−0.30 × τa − 0.19, 0.20 × τa + 0.55]. This implied that at least 71% of the AOD bias fell within this range. Additionally, in regions of low aerosol concentration (AOD < 0.6), the retrieval bias decreased with increasing AOD, whereas the bias increased with increasing AOD when AOD > 0.8. As shown in Figure 3e, the EE of the offshore region was defined as [−0.21 × τa − 0.11, 0.21 × τa + 0.77], which contained 64% of the AOD bias. At the same time, the results indicate that the retrieval bias gradually increased with the AOD value.

Figure 3c,f illustrate the spatial distribution of the mean percentage error of LDA AOD for each AERONET site in the inland and offshore regions, respectively. Sites with more than 100 paired samples were included to ensure the reliability of the bias analysis. Significant negative bias (~85%) was observed in the northern and eastern offshore parts of the study area; slight negative bias (~30%) was seen in Indonesia and Tibet, China; and positive bias was found in Southeast Asia and the coastal areas of East Asia.

#### 3.1.3. Temporal Validation

**Figure 4:**
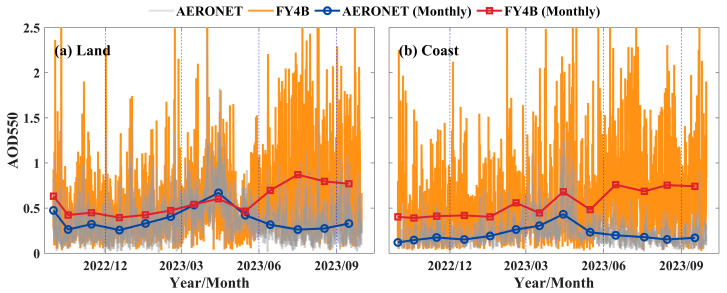
Time series of 10 min (solid line) and monthly average AOD (solid line with marked points) for FY4B and AERONET, October 2022–September 2023. (**a**) Inland areas and (**b**) offshore areas.

To analyze the effect of observation time on the accuracy of LDA AOD, the products were divided into different time scales based on the observation time of the AGRI data for time series analysis and validation. The time series of 10-min (solid line) and monthly AOD averages (solid line with labeled points) for all available pairs of LDA AOD and AERONET site AOD matches in the inland and offshore regions are shown in Figure 4. As shown in Figure 4a, the monthly mean LDA AOD over the inland region was consistent with AERONET AOD measurements at moderate aerosol loading levels (AOD ≈ 0.5), such as in March, April, and May 2023. However, when the AERONET AOD was low (AOD < 0.5), the LDA AOD appeared to be overestimated, as observed in October and November 2022 and August and September 2023. In the offshore region, the LDA AOD continued to be overestimated, especially from June through September 2023, as shown in Figure 4b.

**Figure 5:**
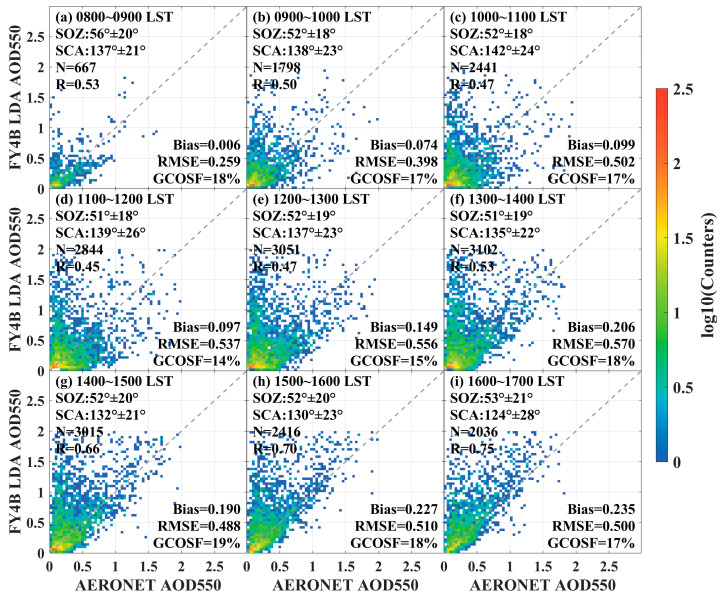
Scatter plots of FY4B LDA AOD 550 versus AERONET observations in the inland region for different time periods. (**a**) 8:00~9:00, (**b**) 9:00~10:00, (**c**) 10:00~11:00, (**d**) 11:00~12:00, (**e**) 12:00~13:00, (**f**) 13:00~14:00, (**g**) 14:00~15:00, (**h**) 15:00~16:00, and (**i**) 16:00~17:00.

In this paper, the product was divided into nine time periods during the day for time series analysis and validation based on the observation time of the data. The observation time of the AGRI sensor was in Universal Time Coordinated (UTC). For the purposes of validation in this study, it is converted to local standard time (LST), which is Beijing Standard Time. The validation scatter plots for different time periods in the inland region are shown in Figure 5. The inland region shows unstable algorithm performance due to the large difference in R and RMSE values in the nine periods. Based on the validation results in the inland region, it was observed that the bias in the morning was smaller than in the afternoon. The figure also showed that the solar zenith angle (SOZ) changed with the time of day and affected the accuracy of the LDA AOD algorithm.

**Figure 6:**
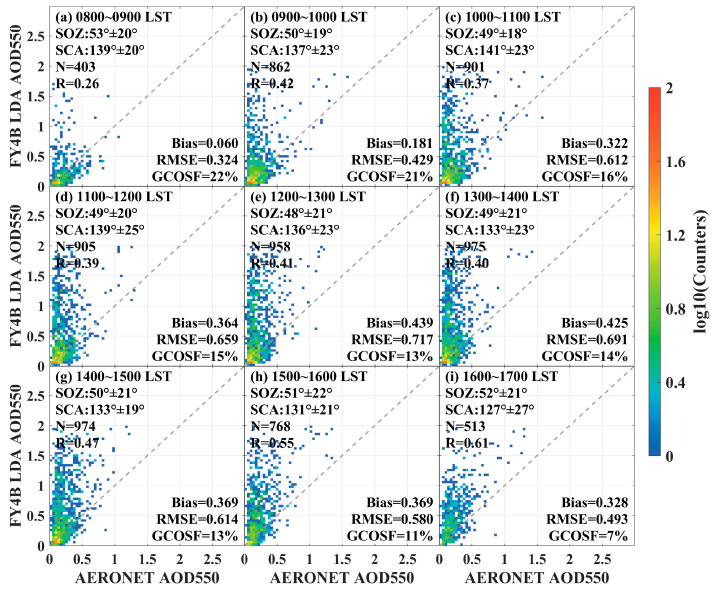
Scatter plots of FY4B LDA AOD 550 versus AERONET observations in the coastal region for different time periods. (**a**) 8:00~9:00, (**b**) 9:00~10:00, (**c**) 10:00~11:00, (**d**) 11:00~12:00, (**e**) 12:00~13:00, (**f**) 13:00~14:00, (**g**) 14:00~15:00, (**h**) 15:00~16:00, and (**i**) 16:00~17:00.

The validation scatter plots for different time periods in the coastal region are shown in Figure 6. In the offshore region, the LDA AOD achieved a maximum R value of 0.61 during the 16:00–17:00 LST period and a minimum R-value of 0.26 during the 8:00–9:00 LST period, indicating unstable algorithm performance due to the large difference in R-values. The maximum RMSE of LDA AOD reached 0.717, while the minimum RMSE was 0.324 across the nine time periods, with retrieval errors varying over time. The worst performance of LDA AOD occurred between 11:00 and 14:00, with Bias ranging from 0.364 to 0.439, RMSE from 0.659 to 0.717, R from 0.39 to 0.41, and GCOSF from 13% to 15%. The LDA AOD retrieval algorithm showed tendencies for both overestimation and underestimation, with overall worse performance in the offshore region compared to the inland region.

Since the scattering angle, satellite zenith angle, and solar zenith angle participate in the calculation of surface reflectance and total transmittance, they thus affect aerosol retrieval. The scattering angle could be calculated by Equation (6) [45].

(6) $$\Theta =\arccos\left(-\cos\theta_{s}\cos\theta_{v}+\sin\theta_{s}\sin\theta_{v}\cos\phi \right)$$

where, Θ is the scatter angle; $\theta_{s}$ is the solar zenith angle; $\theta_{v}$ is the satellite zenith angle; and ϕ is the relative azimuth. 

This paper investigated the dependence of LDA AOD on the solar zenith angle, the satellite zenith angle, and the scatter angle. The average value of 550 nm AOD deviation in each angular interval was taken to explore the response properties of LDA AOD retrieval errors to the satellite zenith angle, the solar zenith angle, and the scattering angle. Among them, the scattering angle is calculated based on FY-4B/AGRI, the satellite zenith angle is measured by FY-4B/AGRI, and the solar zenith angle is measured by AERONET. To ensure the reliability of the validation analysis results, the angular information of the LDA 550 nm AOD was filtered, retaining only solar zenith angles and satellite zenith angles smaller than 80°.

**Figure 7:**
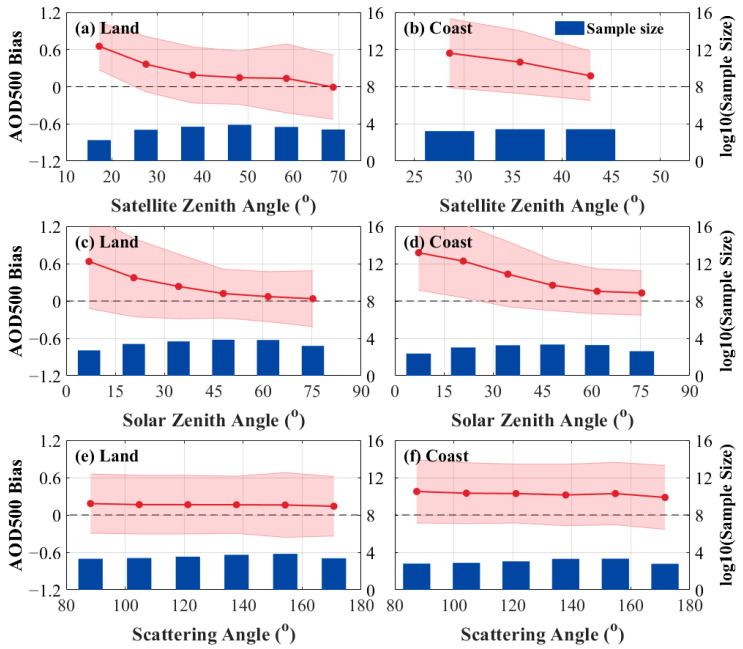
Angular dependence of the AOD 550 bias ($Bias=\tau_{LDA}-\tau_{\mathrm{aeronet}}$) on the number of match points (blue bars) between FY4B and AERONET: (**a**) zenith angle of satellite observations in inland and (**b**) offshore regions; (**c**) AERONET solar zenith angle of observations in inland and (**d**) offshore stations as well as AERONET; and (**e**) scattering angle of inland and (**f**) offshore observations.

The dependence analysis plots of the aerosol bias on the angles are shown in Figure 7. The red solid line indicates the difference between the FY-4B LDA AOD and the AERONET AOD, while the blue bars represent the number of matched pairs of points between the LDA AOD and the AERONET. The biases are all above the zero scale, indicating that the LDA AOD retrieval values were higher than the AERONET measured values, showing a significant overestimation bias.

As shown in Figure 7a, the uncertainty of the LDA AOD retrieval results varied with the satellite zenith angle. In Figure 7b, the offshore region data in the angular range were sparser, which affects the validation results of LDA AOD with AERONET at higher satellite zenith angles. According to the available results, in the satellite zenith angle range of 25° to 45°, the error gradually decreased with the increasing satellite zenith angle. The satellite zenith angle, as a vital factor in calculating the total transmittance of the radiated signal from the Earth’s surface to the sensor, showed a significant effect on the aerosol product quality in both inland and offshore regions.

From Figure 6c,d, it can be observed that as the solar zenith angle increases, the deviation in AOD decreases in both regions. This suggests that smaller solar zenith angles have a greater effect on the accuracy of LDA AOD, which may be related to the intensity of solar radiation [46,47].

Figure 6e,f showed that the influence of scattering angle changes on AOD bias was more stable. The change in LDA AOD retrieval bias was not significant with increasing scattering angle, with the bias remaining low in the inland region, around 0.2. In the offshore region, the AOD bias remained around 0.4, indicating that the scattering angle had a relatively small influence on LDA AOD in the inland region.

**Figure 8:**
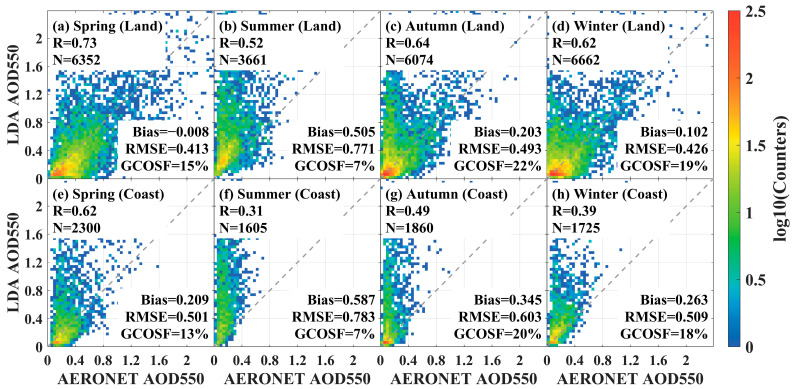
Scatter plots of FY4B LDA AOD 550 and AERONET observations in different seasons. (**a**–**d**) Inland areas; (**e**–**h**) offshore areas.

In this paper, the seasonal variations of LDA AOD between inland and offshore regions in China were explored. As shown in Figure 8a–d, the GCOSF of LDA AOD in inland regions was higher in the fall (22%), compared to the winter (19%) and spring (15%). The retrieval accuracy was the poorest, and the bias was largest in the summer (R = 0.52, Bias = 0.505, RMSE = 0.771, GCOSF = 7%). For offshore AERONET sites, the GCOSF was higher in the fall (20%) compared to the winter (18%) and spring (13%) (Figure 8e–h). The LDA AOD algorithm showed the worst retrieval performance in the summer, with R = 0.31, Bias = 0.587, RMSE = 0.783, and GCOSF = 7%. Both the inland and offshore areas achieved the best performance in the spring. These results indicated that regional and seasonal variations significantly affected LDA AOD retrieval results.

### 3.2. Dependence Analysis

#### 3.2.1. Aerosol Type Dependence Analysis

Due to the differences in particle diameters and the scattering and absorbing capacities of aerosol particles for solar radiation, the accuracy of retrieval results can be affected by the inappropriate aerosol type [48,49,50,51]. The AERONET site provided a large number of aerosol parameters, such as AOD, AE, and Single Scattering Albedo (SSA), which were used to assist in selecting and determining the aerosol model. Additionally, the Ångström Exponent indicated the proportion of small aerosol particles in the atmosphere, which could be analyzed to understand the aerosol’s source, chemical composition, transport path, and its capacity to scatter and absorb solar radiation. The SSA, representing the ratio of the scattering coefficient to the extinction coefficient, was used to differentiate aerosols with varying absorption levels [48]. This research explores the aerosol type dependence of LDA AOD and classifies the aerosol types into Dust (AE < 0.6 and SSA < 0.95), Mixture (0.6 ≤ AE < 1.2), Non-absorbing (NA, AE ≥ 1.2 and SSA ≥ 0.95), Slightly-absorbing (SA, AE ≥ 1.2 and 0.90 ≤ SSA < 0.95), Moderately-absorbing (MA, AE ≥ 1.2 and 0.85 ≤ SSA ≤ 0.90), and Highly-absorbing (HA, AE ≥ 1.2 and SSA ≤ 0.85) [24] by analyzing the studies of Mielonen et al. [39] and Lee et al. [38].

**Figure 9:**
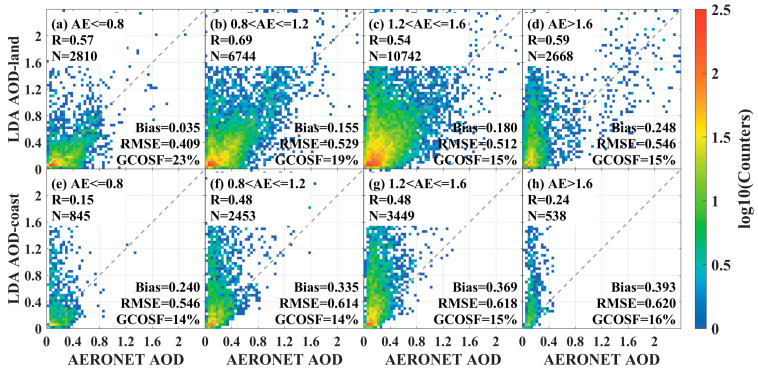
Distribution of FY4B LDA AOD and AERONET observations in inland and offshore regions under different AE conditions. (**a**–**d**) inland areas and (**e**–**h**) offshore areas.

The validation scatter plots of LDA AOD with AERONET measurements in inland and offshore regions under different AE conditions are shown in Figure 9. From Figure 9a, it was observed that in the inland region, the AE had the most matched pairs of points (N = 10,742) in the interval of 1.2 < AE ≤ 1.6, indicating a higher proportion of small-sized aerosol particles in this region. Under the condition of AE > 1.6, the LDA AOD showed significant overestimation with poor retrieval accuracy (Bias = 0.248, RMSE = 0.546, GCOSF = 15%). Conversely, under the condition of AE ≤ 0.8, the retrieval accuracy of the LDA AOD was relatively good (Bias = 0.035, RMSE = 0.409, GCOSF = 23%). These results demonstrated that in the inland region, the higher percentage of small-particle aerosols had a greater impact on retrieval accuracy. In the offshore region, higher AE was also accompanied by poorer retrieval accuracy (Bias = 0.393, RMSE = 0.620, GCOSF = 16% for AE > 1.6), while a lower small-size aerosol particle fraction was accompanied by better accuracy (Bias = 0.240, RMSE = 0.546, GCOSF = 14% for AE ≤ 0.8). This suggested that a higher percentage of small-size aerosol particles was detrimental to retrieval accuracy in both inland and offshore regions.

**Figure 10:**
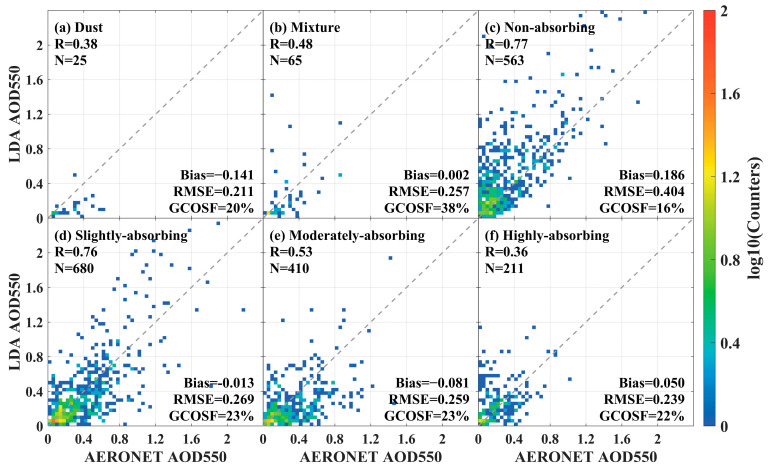
Scatterplot of FY4B LDA AOD 550 with AERONET observations and different aerosol types. (**a**) Dust type; (**b**) Mixture type; (**c**) Non-absorbing type; (**d**) Slightly-absorbing type; (**e**) Moderately-absorbing type; and (**f**) Highly-absorbing type.

The validated scattering results of FY4B LDA 550 nm AOD and AERONET AOD in the inland region under different aerosol types are shown in Figure 10. However, due to the lack of sufficient AE and SSA data at certain sites, the availability of valid data is limited, resulting in a significant reduction in the number of matching samples. As shown in Figure 10, the R ranged from 0.36 to 0.77, Bias ranged from −0.141 to 0.186, RMSE ranged from 0.211 to 0.404, and GCOSF ranged from 16% to 38%. The best accuracy of LDA AOD was obtained when the aerosol type was slightly absorbing, with R = 0.76, N = 680, Bias = −0.013, RMSE = 0.269, and GCOSF = 23%. For dust aerosols, LDA AOD showed a slight negative bias (Bias = −0.141, R = 0.38, RMSE = 0.211, GCOSF = 20%) (Figure 10a). Figure 10d–f shows that as the absorptive capacity of aerosol particles decreased, the agreement between the LDA AOD 550 and AERONET measurements increased (R from 0.36 to 0.76), the bias decreased (0.050 to −0.013), and the GCOSF increased (22% to 23%). These results indicated that SSA had a significant effect on LDA aerosol retrieval over the inland region.

**Figure 11:**
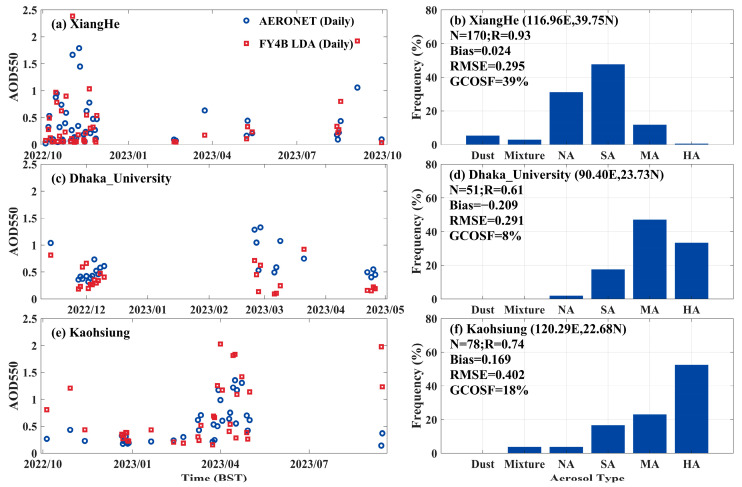
Daily AOD time series of FY4B and AERONET measurements at three AERONET stations for different major aerosol types; the temporal variation AOD (**a**) and aerosol type distribution (**b**) at XiangHe Site, the temporal variation AOD (**c**) and aerosol type distribution (**d**) at Dhaka University Site, and the temporal variation AOD (**e**) and aerosol type distribution (**f**) at Kaohsiung Site.

To further explore the impact of aerosol types on LDA AOD accuracy, this study selected three representative AERONET sites from 72 sites within the study area, each representing a different primary aerosol type, to evaluate LDA AOD, as shown in Figure 11. Figure 11a,b showed that the LDA AOD at the XiangHe site with a slightly absorbing sample had a low bias (Bias = 0.024, RMSE = 0.295), a relatively high GCOSF (39%), and a good correlation (R = 0.93). In Figure 11c,d, at the Dhaka University site, which had moderately-absorbing and highly-absorbing aerosols, the LDA AOD showed a low correlation (R = 0.61) and a significant negative bias (Bias = −0.209), with a GCOSF of only 8%. Additionally, the LDA AOD showed continuous underestimation at this site during December 2022. As shown in Figure 11e,f, the Kaohsiung sites were dominated by highly-absorbing aerosols, and the LDA AOD validation results were the worst, with the highest RMSE (0.402) and a GCOSF of 18%. Therefore, the results indicated that moderately absorbing aerosols and strongly absorbing aerosols significantly impacted LDA AOD retrieval errors, while non-absorbing and slightly absorbing aerosols had a smaller effect on LDA AOD bias among three sites.

#### 3.2.2. Surface Type Dependence Analysis

Different surface cover types affected the accuracy of aerosol retrieval by directly influencing the estimation of surface reflectance and influencing the determination of aerosol type. The normalized vegetation index (NDVI) could be an important parameter for referring to surface cover type in a region, but classification based on NDVI alone did not provide reliable results. Therefore, the MODIS land cover product (MCD12Q1) was used to accurately classify surface types and accordingly analyze the performance of the LDA AOD algorithm on different surface covers. This study selects nine land cover types to analyze the surface type dependence of LDA AOD.

**Figure 12:**
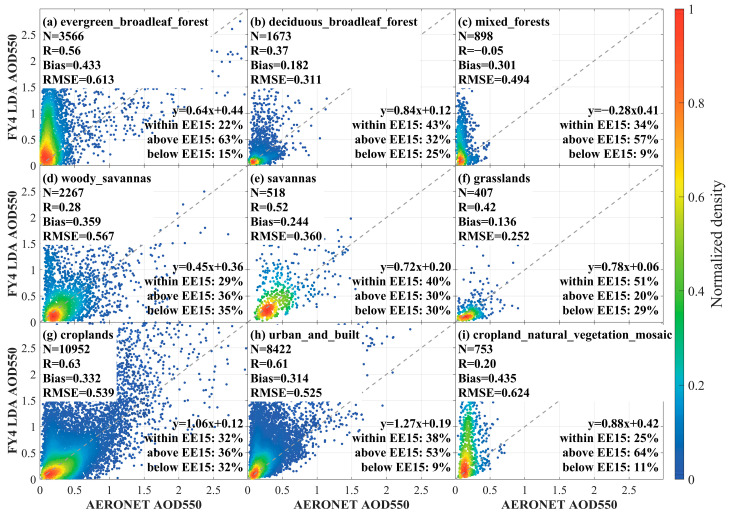
Scatterplot of LDA AOD 550 nm versus AERONET AOD measurements under different land use types obtained by the MCD12Q1 product. (**a**) broadleaf evergreen forest; (**b**) broadleaf deciduous forest; (**c**) mixed forest; (**d**) woody savanna; (**e**) savanna; (**f**) grassland; (**g**) agricultural land; (**h**) urban and built-up areas; (**i**) agricultural land/natural vegetation.

The difference in the performance of the LDA AOD algorithm on these different land cover types is shown in Figure 12. As shown in Figure 12a–c, in the forest land type, the LDA AOD algorithm was easily underestimated and showed the worst performance in mixed forest (R = −0.05, Bias = 0.301, RMSE = 0.494, within EE15 = 34%). Figure 12d,e showed the performance of LDA AOD under the woody savanna type and the tropical savanna type. It was observed that LDA AOD appeared underestimated in both savanna type coverage areas, especially in the woody savanna type, where the linear fit of LDA AOD to the matched point pairs of the site data was y = 0.45x + 0.36. As shown in Figure 12f, LDA AOD appeared underestimated in the grassland area (R = 0.42, Bias = 0.136, RMSE = 0.252, within EE15 = 51%). Additionally, Figure 12g showed that the LDA AOD algorithm appears to be overestimated in the cropland region (R = 0.63, Bias = 0.332, RMSE = 0.539, within EE15 = 32%). Figure 12h indicated that the LDA AOD was overestimated in urban and built areas (R = 0.61, Bias = 0.314, RMSE = 0.525, within EE15 = 38%). Figure 12i showed that the LDA AOD in the “cropland natural vegetation mosaic” area had a poor performance, with a Bias of 0.435, RMSE = 0.624, R = 0.20, and within EE15 = 25%.

In summary, LDA AOD retrievals on dark target areas (e.g., broadleaf evergreen and mixed forests) were usually underestimated, while LDA AOD retrievals on bright surfaces (e.g., urban and built-up areas) were easy to overestimate.

**Figure 13:**
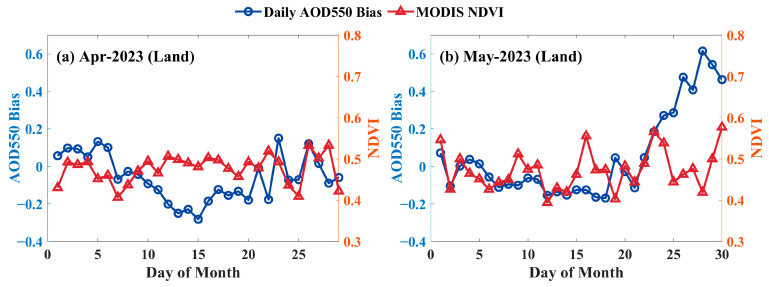
Time series (solid line with markers) of daily mean AOD 550 nm bias (shown by the left *y*-axis, $Bias=\tau_{LDA}-\tau_{AERONET}$), and MODIS NDVI (right *y*-axis) for the inland region. (**a**) April, (**b**) May.

The retrieval deviations are associated with the land surface vegetation conditions, which has been confirmed in the analysis of the relationship between AHI AOD retrieval quality and NDVI [52]. To explore the correlation between the daily mean retrieval deviations of LDA AOD and the MODIS NDVI, an analysis was conducted between the daily mean deviations ($Bias=\tau_{LDA}-\tau_{AERONET}$) and MODIS NDVI during April and May 2023 in inland regions. The results are shown in the line graph in Figure 13, with Figure 13a representing April and Figure 13b representing May. In Figure 13a, it can be observed that from 13 April to 20 April, the retrieval deviations remained relatively stable, with minimal changes in NDVI during this period. However, after 20 April, significant fluctuations in NDVI were observed, corresponding to notable changes in retrieval deviations. In Figure 13b, it can be seen that from 1 May to 8 May, the trends in LDA AOD deviations and NDVI were relatively consistent. From 23 May onwards, the LDA AOD deviations increased significantly, accompanied by dramatic fluctuations in NDVI. Therefore, it can be inferred that relatively stable NDVI contributes to improving the consistency between LDA AOD and AERONET measurements.

### 3.3. Comparison with Other Sensors

**Figure 14:**
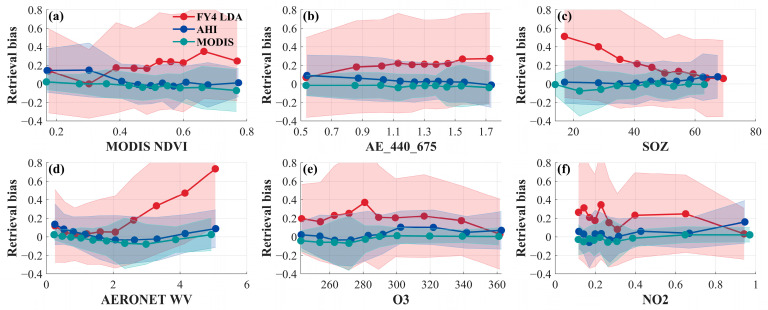
Retrieval error bias ($Bias=\tau_{LDA}-\tau_{AERONET}$) dependence analysis of LDA AOD, AHI AOD, and MODIS AOD. (**a**) MODIS NDVI, (**b**) AE index, (**c**) solar zenith angle, (**d**) AERONET water vapor content, (**e**) ozone concentration, and (**f**) NO_2_ concentration.

To investigate the dependence of LDA AOD retrieval errors ($Bias=\tau_{LDA}-\tau_{\mathrm{aeronet}}$) on different aerosol conditions, six parameters were selected: MODIS NDVI, AE, AERONET solar zenith angle (SOZ), AERONET water vapor (WV), ozone (O_3_), and nitrogen dioxide (NO_2_). The deviation of the three datasets, LDA AOD, AHI AOD, and MODIS AOD, from the AERONET AOD data with respect to these influence parameters is shown in Figure 14.

As seen in Figure 14a, the influence of MODIS NDVI on MODIS AOD (green line) was small. The influence of NDVI on LDA AOD (red line) is more pronounced at lower NDVI values. As NDVI increases, the effect of MODIS NDVI on AHI AOD (blue line) retrieval bias diminishes and approaches zero. Figure 14b shows that the MODIS AOD is almost unaffected by the AE. As the AE increases, the positive bias in LDA AOD tends to rise, while the retrieval bias in AHI AOD gradually decreases. As illustrated in Figure 14c, the retrieval bias for AHI AOD and MODIS AOD changes slightly with an increase in the SOZ. The influence of the SOZ on LDA AOD is most significant at lower angles, especially between 20° and 30°. Beyond this range, the retrieval bias tends to decrease as the zenith angle increases.

In Figure 14d, the retrieval bias in MODIS AOD and AHI AOD initially decreases with increasing water vapor content, followed by an upward trend. The retrieval bias for LDA AOD significantly increases with higher water vapor content, suggesting incomplete removal of water vapor effects during the LDA AOD retrieval process. This necessitates further adjustment of the algorithm to account for water vapor influence and improve retrieval accuracy.

Figure 14e demonstrates the relationship between retrieval bias for LDA AOD, AHI AOD, and MODIS AOD with ozone concentration. It can be observed that with increasing ozone concentration, the retrieval error for LDA AOD first increases and then decreases. The errors for AHI AOD and MODIS AOD increase with rising ozone concentrations, and then stabilize. As shown in Figure 14f, the LDA AOD error fluctuates significantly within the NO_2_ concentration range of 0.2 to 0.4. Subsequently, with further increases in NO_2_ concentration, the bias first rises and then decreases. AHI AOD and MODIS AOD exhibit a small negative bias at low NO_2_ concentrations, which gradually shifts to a positive bias as the concentration increases.

**Figure 15:**
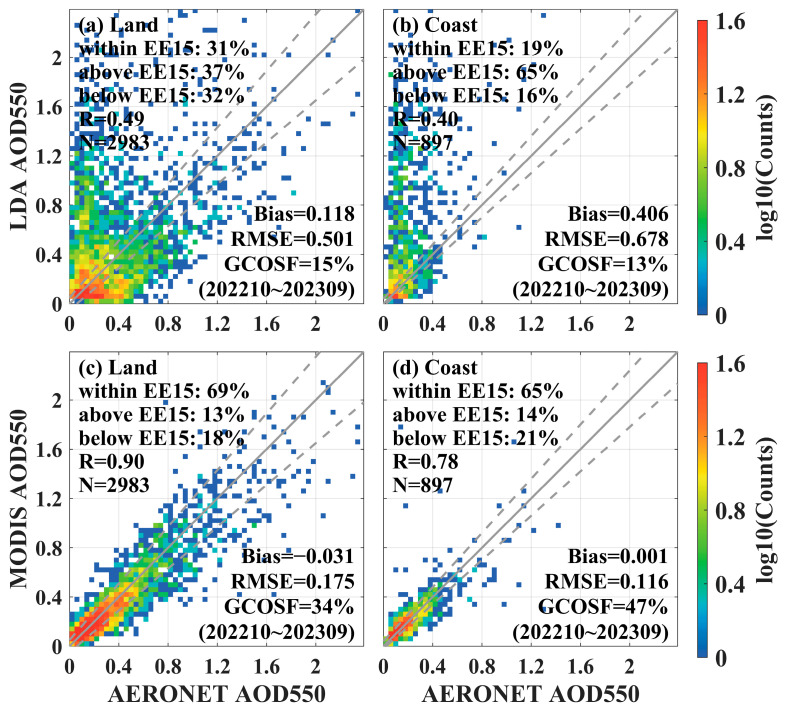
Validation results of FY4B LDA 550 nm AOD and MODIS 550 nm AOD with AERONET observations in the inland region (**a**,**c**) and offshore region (**b**,**d**), respectively.

The results of the accuracy validation of LDA AOD and MODIS AOD with AERONET data are shown in Figure 15. As seen in Figure 15a,c, the within EE15 (31%), correlation coefficient (R = 0.49), and GCOSF percentage (15%) of LDA AOD in the inland region were significantly lower than those of MODIS AOD (within EE15 = 69%, R = 0.90, GCOSF = 34%), while the Bias (0.118) and RMSE (0.501) were both higher than those of MODIS AOD (Bias = −0.031, RMSE = 0.175). The precision difference between LDA AOD and MODIS AOD in the offshore region was similar to that in the inland region, as shown in Figure 15b,d, with R (0.40 vs. 0.78), Bias (0.406 vs. 0.001), RMSE (0.678 vs. 0.116), within EE15 (19% vs. 65%), and GCOSF (13% vs. 47%). However, LDA AOD showed significant overestimation in the low aerosol concentration region. In summary, the accuracy of LDA AOD was worse than that of MODIS AOD products in both inland and offshore regions.

From the results of the angular dependence analysis in Figure 7, it was evident that LDA AOD was susceptible to the influence of the solar zenith angle and the angular magnitude of the satellite zenith angle. LDA AOD products showed a large positive bias when the solar zenith angle observed by AERONET was less than 45° and when the angle of the satellite zenith angle measured by FY-4B/AGRI was less than 45°. Therefore, this study controlled the solar and satellite angles of LDA AOD, removing sample points with angles less than 45° and comparing them with MODIS AOD for validation. However, due to the drastic reduction in matched pairs of points in the offshore region after angle control, only the validation results of the inland region were explored after the angle control.

**Figure 16:**
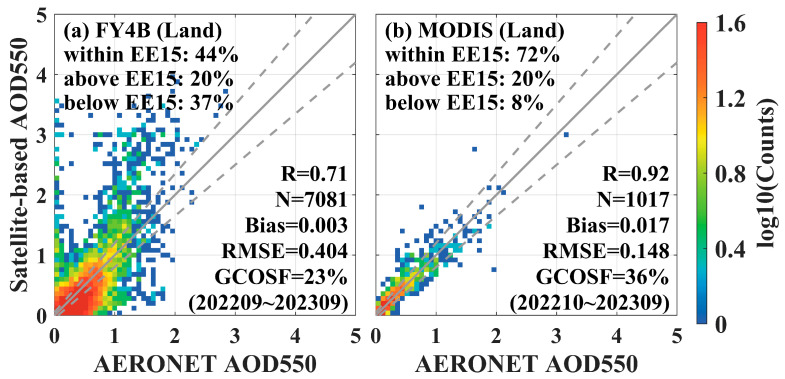
Validated scatterplots of LDA AOD, MODIS AOD, and AERONET data for the inland region after removing angular effects (removing SOZ_AERONET < 45° and SAZ_FY4B < 45°). (**a**) LDA AOD, (**b**) MODIS AOD.

The scatterplot of the validation of LDA AOD with MODIS AOD and AERONET measurements in the inland region after angular control is shown in Figure 16. After avoiding the effect of smaller solar zenith angles and satellite zenith angles on the performance of the LDA AOD algorithm, the accuracy of the LDA AOD retrieval over the inland region significantly improved, with R improving from 0.49 to 0.71, within EE15 improving from 31% to 44%, Bias decreasing from 0.118 to 0.003, and RMSE decreasing from 0.501 to 0.404. GCOSF improved from 15% to 23%. However, LDA AOD overestimation still existed at higher aerosol concentrations in the inland region, as shown in Figure 15a and Figure 16a. MODIS AOD retrieval accuracy was also improved, as shown in Figure 16b (within EE15 = 72%, R = 0.92, Bias = 0.017, RMSE = 0.148, and GCOSF = 36%).

## 4. Discussion and Application Analysis

This study evaluates the retrieval performance of the AGRI LDA AOD algorithm in China from 30 September 2022, to 30 September 2023. The annual validation results reveal a moderately consistent relationship between LDA AOD and AERONET measurements (R = 0.55, Bias = 0.328, RMSE = 0.533, within EE15 = 34%). The average yearly LDA AOD distribution shows a clear overestimation in the coastal and high-altitude localities, which can also be observed in the GEOS-16 ABI AOD [53]. Additionally, LDA AOD retrievals are underestimated over dark surfaces and overestimated over bright surfaces, such as urban and built areas, indicating limitations in LDA AOD retrieval over bright surfaces. LDA AOD is derived using the Dark Target (DT) method, which relies on empirical relationships to estimate surface reflectance and will introduce biases, particularly in bright surface areas, reducing the accuracy of the results. The analysis showed a high correlation between LDA AOD bias and MODIS NDVI, indicating that surface cover type significantly impacts the algorithm’s performance. To improve accuracy, it is necessary to consider the influence of surface cover types and modify the method for obtaining surface reflectance.

The 72 AERONET stations were divided into 50 inland and 22 offshore stations based on their distance from the coastline. Validation results show that LDA retrievals are more reliable in inland than offshore regions, likely due to the influence of water vapor content and marine aerosols. The retrieval bias dependence analysis of LDA AOD indicates a significant impact of water vapor on LDA AOD. The relationship between water vapor content and AOD retrievals suggests that higher water vapor content can lead to increased retrieval errors. Enhancing the algorithm to better account for water vapor effects is essential for improving retrieval performance.

The impact of solar zenith angle (SOZ) and satellite zenith angle (SAZ) on AOD retrieval is significant. Smaller SOZ and SAZ typically result in larger retrieval biases. This is because lower solar and satellite angles increase the radiative path, thereby enhancing the scattering effect and increasing the complexity of AOD retrieval. Consequently, adjustments to the algorithm are necessary to improve retrieval accuracy in low-angle regions and during times of day with low SOZ and SAZ [46,47], such as in the morning and late afternoon. Moreover, the seasonal performance differences of the algorithm may be related to aerosol types. The chosen aerosol types may not represent all seasonal aerosol information, impacting the retrieval accuracy in AOD retrieval [50,51]. Considering the regional and seasonal changes in aerosol characteristics, the selection of aerosol types in the adjustment region can further improve the accuracy of the AOD retrieval algorithm.

LDA AOD is more suitable for AOD retrieval in inland areas, particularly yielding reliable results in northeastern China. Time series validation indicates that, whether in inland or coastal regions, LDA AOD data in the morning are more accurate than in the afternoon, with the smallest deviation from AERONET measurements observed between 8:00 and 9:00 AM. This suggests that morning observations are more reliable due to more stable atmospheric conditions and less interference from other variables. The LDA algorithm is more effective for monitoring aerosol changes in China during the spring, and better performance occurred in areas with high solar zenith and satellite zenith angles. A higher proportion of small particle aerosols and stronger aerosol absorption capabilities negatively impact retrieval accuracy. Error dependence analysis reveals that LDA AOD biases are more stable with lower water vapor content. High water vapor content can cause significant interference in the retrieval process, leading to inaccuracies. Therefore, regions and times with lower water vapor content are more suitable for using LDA AOD products. Additionally, it is found that NDVI has a strong correlation with AOD retrieval performance [52]. Areas with stable NDVI characteristics achieve more reliable LDA AOD results. The NDVI reflects vegetation cover and density and affects the calculation of surface reflectance. Stable NDVI values indicate consistent vegetation cover and reduce the variability of surface reflectance, which can help obtain a more accurate AOD.

In conclusion, although LDA AOD shows great potential for AOD retrieval, especially in inland areas and at certain times of the day and year, continuous improvement is needed to improve its applicability.

## 5. Conclusions

A comprehensive analysis and validation were conducted for the land aerosol products of the Chinese geostationary satellite Fengyun-4B. The impact of differences in observation time, angle dependence, aerosol type, and surface type on the performance of the AGRI official land aerosol product AOD retrieval algorithm was investigated. The main findings are as follows:

(1) Validation results based on AERONET ground-based data indicated that LDA AOD is moderately consistent with ground-based measurements (R = 0.55, Bias = 0.328, RMSE = 0.533, within EE15 = 34%). The LDA AOD in the southern coastal and western plateau regions of China is easy to overestimate.
(2) Analysis results in inland and coastal areas indicate that LDA AOD exhibits significant overestimation in coastal regions, and the algorithm’s retrieval performance in coastal (R = 0.43, Bias = 0.334, RMSE = 0.597, GCOSF = 15%) regions is inferior to that in inland areas (R = 0.60, Bias = 0.163, RMSE = 0.509, GCOSF = 17%).
(3) Error analysis revealed that the retrieval bias of LDA AOD was highly dependent on the satellite zenith angle and solar zenith angle, especially in low-angle regions where the bias changed significantly with increasing angle. Additionally, the retrieval bias of LDA AOD was greatly influenced by water vapor content.
(4) Analysis of aerosol type dependence indicated that LDA AOD was significantly affected by the absorption and scattering characteristics of aerosol particles. Moderately absorbing and strongly absorbing aerosols had a greater impact on the retrieval bias of LDA AOD, while non-absorbing and slightly absorbing aerosols had a smaller effect on the bias of LDA AOD.
(5) The LDA AOD algorithm performs more reliably in inland areas, regions with stable NDVI, or conditions with lower water vapor content. Analysis results of aerosol type dependence indicate that the LDA AOD algorithm is more accurate when the proportion of fine particulate aerosols is lower or the aerosol absorption capacity is weaker. Additionally, during the spring or morning hours, LDA AOD shows greater consistency with AERONET measurements when atmospheric conditions are more stable.

Future improvements to the LDA AOD retrieval algorithm should focus on refining the determination of surface reflectance, mitigating the interference of water vapor content on data, and selecting more accurate aerosol models that account for regional and seasonal variations to enhance the algorithm’s accuracy.

## Data Availability

The Fengyun-4B is available from the NSMC’s official website (http://satellite.nsmc.org.cn/, accessed on 5 March 2024); the Himawari-9/AHI data is available from JAXA’s official website (https://www.eorc.jaxa.jp/ptree/, 29 March 2024); The MODIS data can be obtained from NASA’s official website (https://ladsweb.modaps.eosdis.nasa.gov/, 14 March 2024); and The AERONET measurements are available from NASA’s official website (https://aeronet.gsfc.nasa.gov, 26 March 2024).

## References

[1] Liu X., Chen Q., Che H., Zhang R., Gui K., Zhang H., Zhao T. (2016). Spatial distribution and temporal variation of aerosol optical depth in the Sichuan basin, China, the recent ten years. Atmos. Environ..

[2] Rosenfeld D., Dai J., Yu X., Yao Z., Xu X., Yang X., Du C. (2007). Inverse relations between amounts of air pollution and orographic precipitation. Science.

[3] Lin C., Liu G., Lau A.K.H., Li Y., Li C., Fung J.C.H., Lao X.Q. (2018). High-resolution satellite remote sensing of provincial PM2. 5 trends in China from 2001 to 2015. Atmos. Environ..

[4] Pope III C.A., Burnett R.T., Krewski D., Jerrett M., Shi Y., Calle E.E., Thun M.J. (2009). Cardiovascular mortality and exposure to airborne fine particulate matter and cigarette smoke: Shape of the exposure-response relationship. Circulation.

[5] Twomey S. (1991). Aerosols, clouds and radiation. Atmos. Environment. Part A Gen. Top..

[6] Yu L., Zhang M., Wang L., Lu Y., Li J. (2021). Effects of aerosols and water vapour on spatial-temporal variations of the clear-sky surface solar radiation in China. Atmos. Res..

[7] Li Y., Xue Y., Guang J., She L., Fan C., Chen G. (2018). Ground-level PM2. 5 concentration estimation from satellite data in the Beijing area using a specific particle swarm extinction mass conversion algorithm. Remote Sens..

[8] Holben B.N., Eck T.F., Slutsker I.a., Tanré D., Buis J., Setzer A., Vermote E., Reagan J.A., Kaufman Y., Nakajima T. (1998). AERONET—A federated instrument network and data archive for aerosol characterization. Remote Sens. Environ..

[9] Holben B.N., Tanré D., Smirnov A., Eck T., Slutsker I., Abuhassan N., Newcomb W., Schafer J., Chatenet B., Lavenu F. (2001). An emerging ground-based aerosol climatology: Aerosol optical depth from AERONET. J. Geophys. Res. Atmos..

[10] Sun X., Sun L., Sun Y., Zhang J., Fan X., Ma C. (2024). Inversion of Aerosol Optical Depth: Incorporating Multi-Model Approach. IEEE Trans. Geosci. Remote Sens..

[11] Bao F., Gu X., Cheng T., Wang Y., Guo H., Chen H., Wei X., Xiang K., Li Y. (2016). High-spatial-resolution aerosol optical properties retrieval algorithm using Chinese high-resolution earth observation satellite I. IEEE Trans. Geosci. Remote Sens..

[12] Jin S., Zhang M., Ma Y., Gong W., Chen C., Yang L., Hu X., Liu B., Chen N., Du B. (2021). Adapting the dark target algorithm to advanced MERSI sensor on the FengYun-3-D satellite: Retrieval and validation of aerosol optical depth over land. IEEE Trans. Geosci. Remote Sens..

[13] Fan X., Qu Y. (2019). Retrieval of high spatial resolution aerosol optical depth from HJ-1 A/B CCD data. Remote Sens..

[14] Sun L., Wei J., Bilal M., Tian X., Jia C., Guo Y., Mi X. (2015). Aerosol optical depth retrieval over bright areas using Landsat 8 OLI images. Remote Sens..

[15] Hsu N., Lee J., Sayer A., Kim W., Bettenhausen C., Tsay S.C. (2019). VIIRS Deep Blue aerosol products over land: Extending the EOS long-term aerosol data records. J. Geophys. Res. Atmos..

[16] Kaufman Y.J., Wald A.E., Remer L.A., Gao B.-C., Li R.-R., Flynn L. (1997). The MODIS 2.1-/spl mu/m channel-correlation with visible reflectance for use in remote sensing of aerosol. IEEE Trans. Geosci. Remote Sens..

[17] Li L., Franklin M., Girguis M., Lurmann F., Wu J., Pavlovic N., Breton C., Gilliland F., Habre R. (2020). Spatiotemporal imputation of MAIAC AOD using deep learning with downscaling. Remote Sens. Environ..

[18] Xie Y., Li Z., Guang J., Hou W., Salam A., Ali Z., Fang L. (2021). Aerosol optical depth retrieval over south Asia using FY-4A/AGRI data. IEEE Trans. Geosci. Remote Sens..

[19] Su X., Wang L., Zhang M., Qin W., Bilal M. (2021). A high-precision aerosol retrieval algorithm (HiPARA) for advanced Himawari imager (AHI) data: Development and verification. Remote Sens. Environ..

[20] Pope Iii C.A., Burnett R.T., Thun M.J., Calle E.E., Krewski D., Ito K., Thurston G.D. (2002). Lung cancer, cardiopulmonary mortality, and long-term exposure to fine particulate air pollution. JAMA Netw..

[21] Jiang X., Xue Y., Jin C., Bai R., Sun Y., Wu S. (2022). A Simple Band Ratio Library (BRL) Algorithm for Retrieval of Hourly Aerosol Optical Depth Using FY-4A AGRI Geostationary Satellite Data. Remote Sens..

[22] Su X., Wang L., Cao M., Yang L., Zhang M., Qin W., Cao Q., Yang Y., Li L. (2023). Fengyun 4A Land Aerosol Retrieval: Algorithm Development, Validation, and Comparison with other datasets. IEEE Trans. Geosci. Remote Sens..

[23] Bessho K., Date K., Hayashi M., Ikeda A., Imai T., Inoue H., Kumagai Y., Miyakawa T., Murata H., Ohno T. (2016). An introduction to Himawari-8/9—Japan’s new-generation geostationary meteorological satellites. J. Meteorol. Soc. Jpn. Ser. II.

[24] Wang W., Mao F., Pan Z., Gong W., Yoshida M., Zou B., Ma H. (2019). Evaluating aerosol optical depth from Himawari-8 with sun photometer network. J. Geophys. Res. Atmos..

[25] Xu W., Wang W., Wang N., Chen B. (2022). A new algorithm for Himawari-8 aerosol optical depth retrieval by integrating regional PM_2.5_ concentrations. IEEE Trans. Geosci. Remote Sens..

[26] Fukuda S., Nakajima T., Takenaka H., Higurashi A., Kikuchi N., Nakajima T.Y., Ishida H. (2013). New approaches to removing cloud shadows and evaluating the 380 nm surface reflectance for improved aerosol optical thickness retrievals from the GOSAT/TANSO-Cloud and Aerosol Imager. J. Geophys. Res. Atmos..

[27] Higurashi A., Nakajima T. (1999). Development of a two-channel aerosol retrieval algorithm on a global scale using NOAA AVHRR. J. Atmos. Sci..

[28] Yoshida M., Kikuchi M., Nagao T.M., Murakami H., Nomaki T., Higurashi A. (2018). Common retrieval of aerosol properties for imaging satellite sensors. J. Meteorol. Soc. Jpn. Ser. II.

[29] Loveland T.R., Reed B.C., Brown J.F., Ohlen D.O., Zhu Z., Yang L., Merchant J.W. (2000). Development of a global land cover characteristics database and IGBP DISCover from 1 km AVHRR data. Int. J. Remote Sens..

[30] Friedl M.A., Sulla-Menashe D., Tan B., Schneider A., Ramankutty N., Sibley A., Huang X. (2010). MODIS Collection 5 global land cover: Algorithm refinements and characterization of new datasets. Remote Sens. Environ..

[31] Friedl M.A., McIver D.K., Hodges J.C., Zhang X.Y., Muchoney D., Strahler A.H., Woodcock C.E., Gopal S., Schneider A., Cooper A. (2002). Global land cover mapping from MODIS: Algorithms and early results. Remote Sens. Environ..

[32] Huete A., Didan K., Miura T., Rodriguez E.P., Gao X., Ferreira L.G. (2002). Overview of the radiometric and biophysical performance of the MODIS vegetation indices. Remote Sens. Environ..

[33] van Leeuwen W.J., Huete A.R., Laing T.W. (1999). MODIS vegetation index compositing approach: A prototype with AVHRR data. Remote Sens. Environ..

[34] Lyapustin A., Wang Y., Korkin S., Huang D. (2018). MODIS collection 6 MAIAC algorithm. Atmos. Meas. Tech..

[35] Lyapustin A., Wang Y., Laszlo I., Kahn R., Korkin S., Remer L., Levy R., Reid J. (2011). Multiangle implementation of atmospheric correction (MAIAC): 2. Aerosol algorithm. J. Geophys. Res. Atmos..

[36] Zhang Z., Wu W., Fan M., Wei J., Tan Y., Wang Q. (2019). Evaluation of MAIAC aerosol retrievals over China. Atmos. Environ..

[37] Ångström A. (1929). On the atmospheric transmission of sun radiation and on dust in the air. Geogr. Ann..

[38] Lee J., Kim J., Song C., Kim S., Chun Y., Sohn B., Holben B. (2010). Characteristics of aerosol types from AERONET sunphotometer measurements. Atmos. Environ..

[39] Mielonen T., Arola A., Komppula M., Kukkonen J., Koskinen J., De Leeuw G., Lehtinen K. (2009). Comparison of CALIOP level 2 aerosol subtypes to aerosol types derived from AERONET inversion data. Geophys. Res. Lett..

[40] Levy R., Remer L., Kleidman R., Mattoo S., Ichoku C., Kahn R., Eck T. (2010). Global evaluation of the Collection 5 MODIS dark-target aerosol products over land. Atmos. Chem. Phys..

[41] Jiang T., Chen B., Chan K.K.Y., Xu B. (2019). Himawari-8/AHI and MODIS aerosol optical depths in China: Evaluation and comparison. Remote Sens..

[42] Huang J., Kondragunta S., Laszlo I., Liu H., Remer L.A., Zhang H., Superczynski S., Ciren P., Holben B.N., Petrenko M. (2016). Validation and expected error estimation of Suomi-NPP VIIRS aerosol optical thickness and Ångström exponent with AERONET. J. Geophys. Res. Atmos..

[43] GCOS (2011). Systematic Observation Requirements for Satellite-Based Products for Climate.

[44] Popp T., De Leeuw G., Bingen C., Brühl C., Capelle V., Chedin A., Clarisse L., Dubovik O., Grainger R., Griesfeller J. (2016). Development, production and evaluation of aerosol climate data records from European satellite observations (Aerosol_cci). Remote Sens..

[45] Levy R.C., Remer L.A., Mattoo S., Vermote E.F., Kaufman Y.J. (2007). Second-generation operational algorithm: Retrieval of aerosol properties over land from inversion of Moderate Resolution Imaging Spectroradiometer spectral reflectance. J. Geophys. Res. Atmos..

[46] Sun Z., Wei J., Zhang N., He Y., Sun Y., Liu X., Yu H., Sun L. (2021). Retrieving High-Resolution Aerosol Optical Depth from GF-4 PMS Imagery in Eastern China. Remote Sens..

[47] Ge B., Li Z., Chen C., Hou W., Xie Y., Zhu S., Qie L., Zhang Y., Li K., Xu H. (2022). An improved aerosol optical depth retrieval algorithm for multiangle directional polarimetric camera (DPC). Remote Sens..

[48] Che H., Qi B., Zhao H., Xia X., Eck T.F., Goloub P., Dubovik O., Estelles V., Cuevas-Agulló E., Blarel L. (2018). Aerosol optical properties and direct radiative forcing based on measurements from the China Aerosol Remote Sensing Network (CARSNET) in eastern China. Atmos. Chem. Phys..

[49] Horowitz H.M., Garland R.M., Thatcher M., Landman W.A., Dedekind Z., Van der Merwe J., Engelbrecht F.A. (2017). Evaluation of climate model aerosol seasonal and spatial variability over Africa using AERONET. Atmos. Chem. Phys..

[50] Wu Y., Zhu J., Che H., Xia X., Zhang R. (2015). Column-integrated aerosol optical properties and direct radiative forcing based on sun photometer measurements at a semi-arid rural site in Northeast China. Atmos. Res..

[51] Dubovik O., Holben B., Eck T.F., Smirnov A., Kaufman Y.J., King M.D., Tanré D., Slutsker I. (2002). Variability of absorption and optical properties of key aerosol types observed in worldwide locations. J. Atmos. Sci..

[52] Wei J., Li Z., Sun L., Peng Y., Zhang Z., Li Z., Su T., Feng L., Cai Z., Wu H. (2019). Evaluation and uncertainty estimate of next-generation geostationary meteorological Himawari-8/AHI aerosol products. Sci. Total Environ..

[53] Fu D., Gueymard C.A., Xia X. (2023). Validation of the improved GOES-16 aerosol optical depth product over North America. Atmos. Environ..

